# Contributions to the Museum of the Baltimore College of Dent. Surg.

**Published:** 1847-10

**Authors:** 


*Contributions to the Museum of the Baltimore College of Dental Surgery.*—A number of very interesting contributions have been made to the museum of the above named institution, within the last few months. Among the number are, a full set of artificial teeth, constructed by the late Mr. Gardette of Philadelphia, about thirty-six years ago. The design and execution of this specimen of dental mechanism, is equal to any we have ever seen, constructed at the period when this was executed; also a denture for the lower jaw, fabricated by the late Dr. Hudson of the same city, about twenty-five years ago, and a double set of porcelain teeth, with artificial gums, mounted on platina plates. This last was constructed in Paris about sixteen years ago. These, together with a number of other specimens, were presented to the college by Dr. E. Townsand of Philadelphia.

In addition to the above, Mr. I. Wooffendale, surgeon dentist of New York, and son of the late Mr. R. Wooffendale, author of a treatise upon the teeth, published in London, in 1783, presented to us for the museum of this institution, a double set of teeth, constructed by his father upwards of sixty years ago. There is also an upper porcelain denture, with artificial gums, presented by Dr. E. Parmly of New York, and manufactured by N. De Chemant, in London, in 1795.

Besides the above named, and numerous other specimens of early dental mechanism, a large number of specimens of morbid dental anatomy, have recently been presented to the college; and among which, are three molar teeth united by exostosis. These were presented to us for the college by Dr. G. E. Hawes of New York. We received about the same time a number of specimens of diseased teeth, beautifully arranged, from Dr. N. Dodge, of the same city, and within the last day or two, from Dr. S. H. Williams, a central incisor of second dentition, with its root reflected up upon the anterior or labial surface of its crown.

Similar accessions are constantly being made to the museum of this institution, and we would take this opportunity to say, that any specimens, either healthy or morbid, of dental anatomy, or of dental mechanism, which members of the profession may feel disposed to contribute, will be thankfully received, and carefully arranged, with the names of the donors attached to them.—*Balt. Ed.*

